# Ontogeny reveals function and evolution of the hadrosaurid dinosaur dental battery

**DOI:** 10.1186/s12862-016-0721-1

**Published:** 2016-07-28

**Authors:** Aaron R. H. LeBlanc, Robert R. Reisz, David C. Evans, Alida M. Bailleul

**Affiliations:** 1 Department of Biology, University of Toronto Mississauga, Mississauga, ON L5L 1C6 Canada; 2 Department of Optics and Photonics, National Central University, Jhongli City, Taoyuan 32001 Taiwan; 3 Department of Natural History, Royal Ontario Museum, 100 Queen’s Park, Toronto, ON Canada; 4 Department of Pathology and Anatomical Sciences, Integrative Anatomy, University of Missouri, Columbia, MO 65201 USA

## Abstract

**Background:**

Hadrosaurid dinosaurs, dominant Late Cretaceous herbivores, possessed complex dental batteries with up to 300 teeth in each jaw ramus. Despite extensive interest in the adaptive significance of the dental battery, surprisingly little is known about how the battery evolved from the ancestral dinosaurian dentition, or how it functioned in the living organism. We undertook the first comprehensive, tissue-level study of dental ontogeny in hadrosaurids using several intact maxillary and dentary batteries and compared them to sections of other archosaurs and mammals. We used these comparisons to pinpoint shifts in the ancestral reptilian pattern of tooth ontogeny that allowed hadrosaurids to form complex dental batteries.

**Results:**

Comparisons of hadrosaurid dental ontogeny with that of other amniotes reveals that the ability to halt normal tooth replacement and functionalize the tooth root into the occlusal surface was key to the evolution of dental batteries. The retention of older generations of teeth was driven by acceleration in the timing and rate of dental tissue formation. The hadrosaurid dental battery is a highly modified form of the typical dinosaurian gomphosis with a unique tooth-to-tooth attachment that permitted constant and perfectly timed tooth eruption along the whole battery.

**Conclusions:**

We demonstrate that each battery was a highly dynamic, integrated matrix of living replacement and, remarkably, dead grinding teeth connected by a network of ligaments that permitted fine scale flexibility within the battery. The hadrosaurid dental battery, the most complex in vertebrate evolution, conforms to a surprisingly simple evolutionary model in which ancestral reptilian tissue types were redeployed in a unique manner. The hadrosaurid dental battery thus allows us to follow in great detail the development and extended life history of a particularly complex food processing system, providing novel insights into how tooth development can be altered to produce complex dentitions, the likes of which do not exist in any living vertebrate.

**Electronic supplementary material:**

The online version of this article (doi:10.1186/s12862-016-0721-1) contains supplementary material, which is available to authorized users.

## Background

**Fig. 1:**
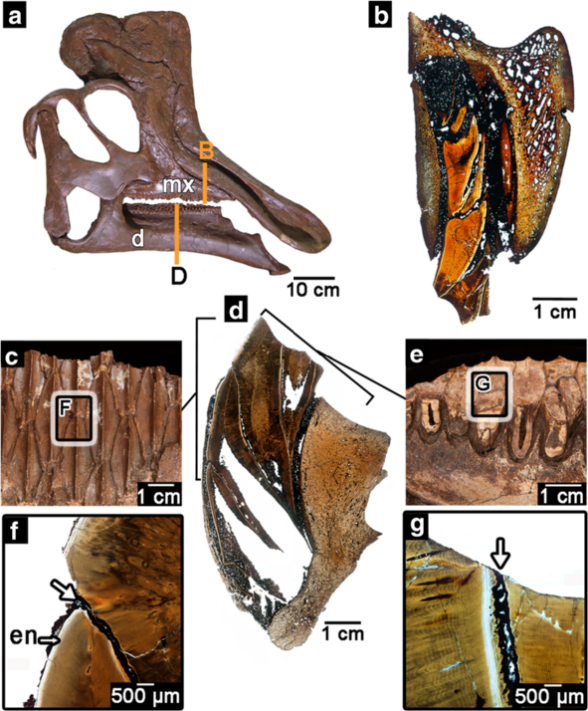
The hadrosaurid dental battery. **a** skull of the hadrosaurid *Corythosaurus* (ROM 00868). Image flipped for the figure. **b** histological thin section through the maxillary dental battery of a hadrosaurid (ROM 00696). **c** lingual view of the dental battery in the lower jaw. **d** histological thin section through the dentary dental battery of the hadrosaurid *Prosaurolophus* (ROM 3500). **e** occlusal surface of the dentary dental battery. **f** closeup image of the intersection between two dentary teeth in a battery, showing the infilling of sediment (*white arrow*) that holds them together. **g** closeup image of the intersection of two teeth along the occlusal surface of a dental battery showing the infilling of sediment that holds the two teeth together. For (**b**) and (**d**), lingual is to the left. d, dentary; en, enamel; mx, maxilla

Hadrosaurid or “duck-billed” dinosaurs were among the most diverse and abundant terrestrial herbivores of the Late Cretaceous [1] and had evolved spectacular adaptations for more efficient grinding and shearing of plant tissues [1–4]. Their success has been linked to the evolution of their complex dental batteries [5, 6], which consist of multiple generations of small, vertically-stacked teeth that interlock with neighbouring teeth [2] (Fig. 1). Some hadrosaurid jaws have up to 300 teeth stacked in 60 tooth positions [1] with multiple functional teeth at each position forming a large, complex grinding surface [2, 7]. This complex chewing surface allowed hadrosaurids to access tough, fibrous plant material by maintaining a constantly replenished oral processing surface with teeth that were at different stages of wear at any given time [5]. Whereas individual teeth appear to have been composed of comparable tissues to those in mammalian teeth [5, 8] the mechanisms that allowed such an unusual dental system to evolve and be maintained have never been investigated and are not understood. We undertook the first ontogenetic study of tooth and tissue interactions in the hadrosaurid dental battery by sectioning large maxillary and dentary batteries and those of embryonic and nestling *Hypacrosaurus* (Additional file 1: Table S1). Each tooth position in the battery preserves up to six teeth at successive ontogenetic stages (Fig. 1), making it possible to reconstruct various stages of dental ontogeny in detail. Thus, hadrosaurid dental batteries offer a unique opportunity to study ontogeny and tooth-to-tooth interactions before and after eruption in a manner that is not possible in any living vertebrate. By comparing the ontogeny of hadrosaurid teeth to that of other archosaurs and mammals, we discovered a unique model of tooth evolution and development that explains how these dentitions—arguably the most complex of any vertebrate- formed and functioned, and discuss their broader significance in vertebrate evolution.

## Methods

Histological thin sections of several amniote taxa (Additional file 1: Table S1) were prepared by first embedding specimens in Castolite AP or Castolite AC polyester resin and placing them under vacuum. One specimen (MOR 559) was embedded in Buehler Epothin resin. Embedded materials were then cut using the Buehler Isomet slow-speed wafer blade saw and the cut surfaces were polished using 600-grit silicon carbide powder. For two specimens (MOR 548, 559), thin wafers were cut using a Buehler Isomet 1000 high-speed wafer blade saw. Specimens were later mounted to frosted plexiglass slides using cyanoacrylate and cut using the Isomet saw. Specimens were then ground down using a Hillquist or a Buehler Ecomet grinding machine and further polished using progressively finer grits of silicon carbide and aluminium oxide powders. The ROM thin sections were imaged using a Nikon DS-Fi camera mounted to a Nikon AZ-100 microscope with NIS Elements BR imaging software registered to D. C. Evans or R. R. Reisz.

## Results

### Histology of a hadrosaurid tooth

**Fig. 2:**
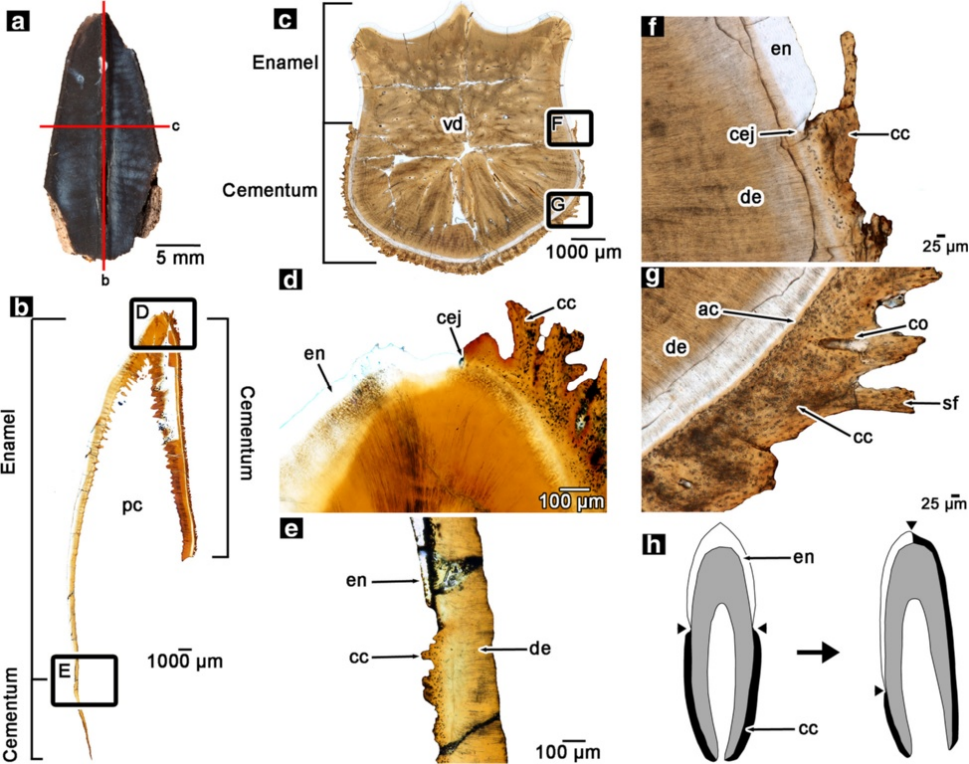
Histology of hadrosaurid teeth. **a** partial hadrosaurid tooth showing planes of section (ROM 58630). **b** isolated wholeview image of a coronal section through an in situ, unerupted maxillary tooth of a hadrosaurid (ROM 00696). **c** isolated wholeview image of a transverse section through an in situ, erupted maxillary tooth of a hadrosaurid (ROM 59042). **d** closup image of the apex of the tooth in (**b**). **e** closeup image of the base of the tooth in (**b**). **f** closeup image of the cemento-enamel junction of the tooth in (**c**). **g** closeup image of the cementum of the tooth in (**c**). **h** illustration of the anatomical differences between a generic amniote tooth (*left*) and a hadrosaurid tooth (*right*). Hadrosaurid teeth exhibit a displacement of the cementum and enamel relative to other amniotes. ac, acellular cementum; cc, cellular cementum; cej, cemento-enamel junction; co, cementeon; de, dentine; en, enamel; pc, pulp cavity; sf, Sharpey’s fibers; vd, vascular dentine

**Fig. 3:**
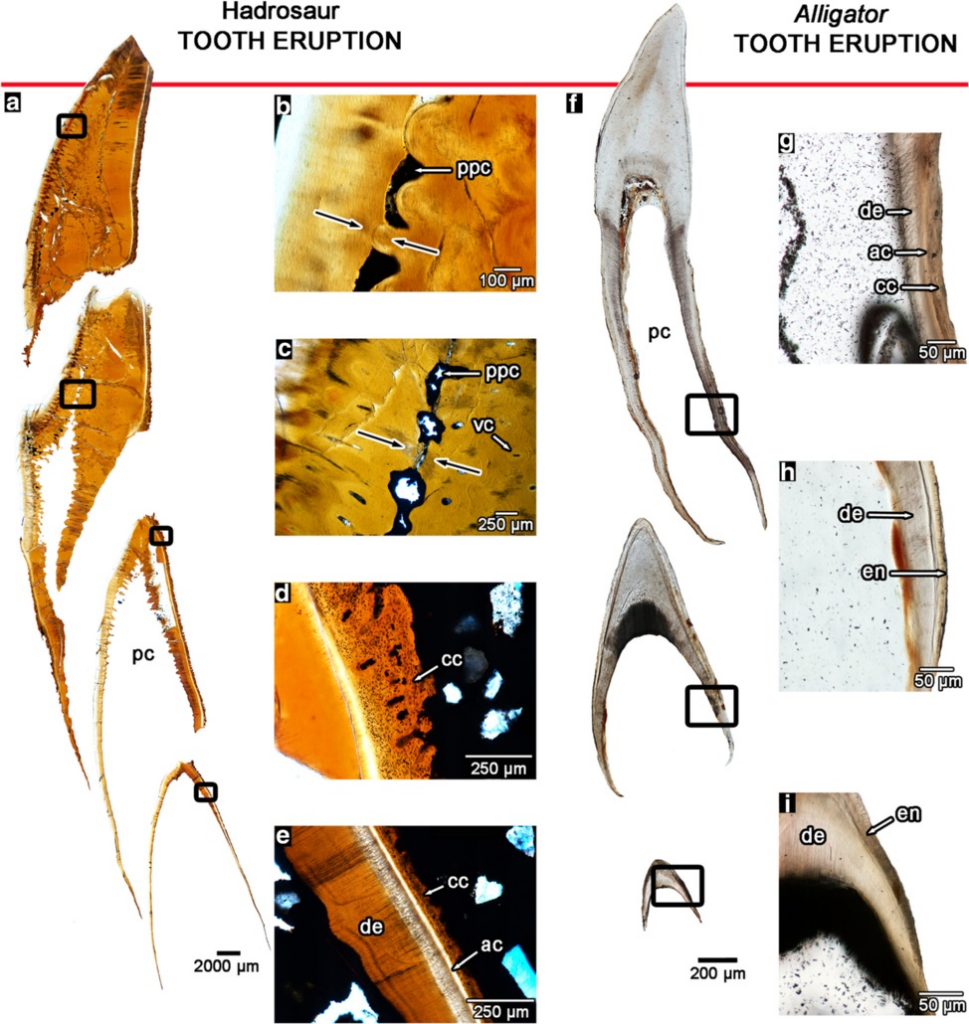
Comparisons of tooth development in hadrosaurids and modern *Alligator*. **a** tooth development sequence in a hadrosaurid (ROM 00696) as seen in histological thin sections. **b** magnified image of the plugged pulp cavity of an erupting hadrosaurid tooth. **c** close-up image of the plugged pulp cavity of an unerupted hadrosaurid tooth. **d** magnified image of the root tissues of an unerupted hadrosaurid tooth. **e** magnified image of the root tissues of a newly formed hadrosaurid tooth. **f** tooth development sequence in a modern hatchling *Alligator* (ROM R6252). **g** magnified image of the root tissues of an erupted *Alligator* tooth. **h** magnified image of an unerupted *Alligator* tooth. **i** magnified image of a newly formed *Alligator* tooth. ac, acellular cementum; cc, cellular cementum; de, dentine; en, enamel; pc, pulp cavity; ppc, plugged pulp cavity; vc, vascular canal

**Fig. 4:**
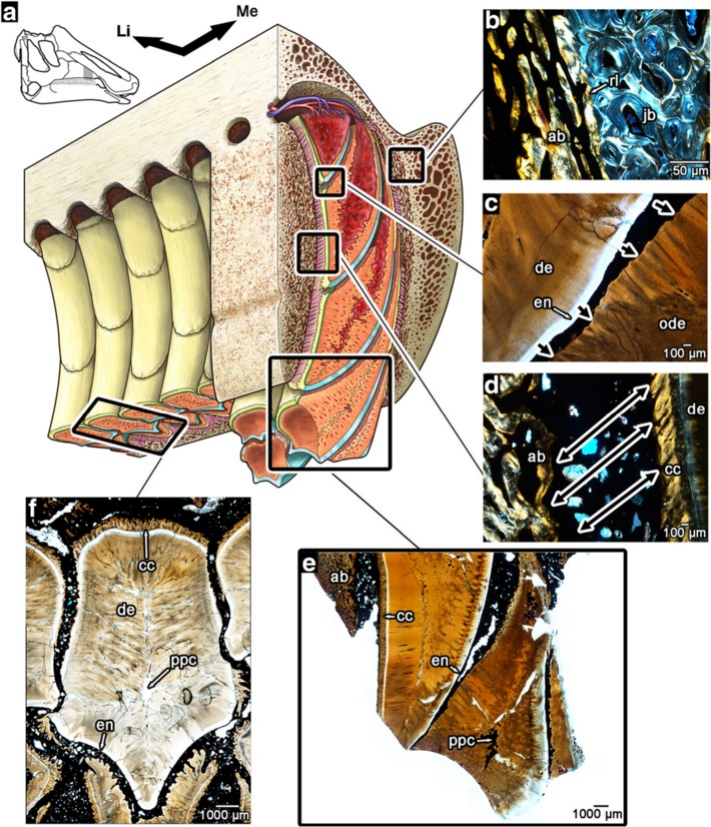
The internal anatomy of the hadrosaurid dental battery. **a** artist’s reconstruction of a portion of the maxillary dental battery, with cutaways in the transverse and coronal planes. For completely labeled reconstruction, see Additional file 3: Figure S2 (illustration by D. Dufault). **b** magnified image of the junction between primary alveolar bone and the remodelled bone of the jaw. **c** magnified image of the resorptive front created by the younger teeth within a vertical stack of teeth (direction of resorption indicated by black arrows). **d** magnified image of the attachment site between the teeth and wall of the socket (direction of periodontal ligament fibers indicated by black arrows). The birefringence in the cellular cementum is caused by the parallel orientations of the Sharpey’s fibers. **e** magnified image of the occlusal end of the dental battery in thin section showing teeth at various stages of wear. **f** image of a tooth within the battery in transverse section. ab, alveolar bone; ac, acellular cementum; cc, cellular cementum; de, dentine; en, enamel; Li, lingual; Me, mesial; pc, pulp cavity; ppc, plugged pulp cavity; rl, reversal line

The histology of the occlusal surfaces of hadrosaurid teeth has been described previously [5, 8], however it is necessary to provide a three-dimensional view of the arrangements of the enamel and dental attachment tissues in unworn teeth in order to understand the development and function of the dental battery. Hadrosaurid teeth consist of enamel- and cementum-covered surfaces that surround a vascularized dentine core (Fig. 2a–c). Unerupted teeth still retain a pulp cavity (Fig. 2b), which would have housed the vital tissues of the tooth, whereas the pulp cavity of erupted teeth is completely replaced by vascular dentine (Fig. 2c). In transverse section, the enamel and cementum surfaces are clearly separate, and cementum never covers the enamel (Fig. 2b–h). In coronal section, the enamel is restricted to the labial surfaces of the maxillary and lingual surfaces of the dentary teeth (Figs. 1, 2, 3 and 4). The cementum and enamel meet at the apex of each tooth and also further towards the root base, past the enamel in maxillary teeth. This arrangement of the enamel and cementum suggests that one of the key differences between hadrosaurid teeth and those of other amniotes is not in the identity of the tissues forming the tooth, but in the re-arrangement of ancestral root and crown tissues, tissues that are also found in other herbivorous and carnivorous dinosaurs [9, 10] (Additional file 2: Figure S1). Instead of forming an enamel cap, which defines the crown in most amniotes, the enamel has shifted to one side of hadrosaurid teeth, with cementum (normally a root tissue [11–14]) occupying the opposite face of the tooth. This arrangement of enamel and cementum was also observed in the sectioned teeth of embryonic and hatchling hadrosaurids (MOR 548, 559). This makes the orientation and function of the “root” and “crown” of a hadrosaurid tooth much different from the condition in a typical amniote, but also has important implications for the timing of the formation of enamel and cementum in hadrosaurid teeth.

### Tooth ontogeny in hadrosaurids

Tooth ontogeny in hadrosaurids can be easily reconstructed using coronal histological sections through a dental battery, which preserve teeth at successive ontogenetic stages (Figs. 3a–e and 4). These sections revealed that, unlike most vertebrates, hadrosaurids did not shed their teeth, and the prevention of normal tooth replacement was a key factor in retaining multiple generations of teeth at each locus. The earliest-forming teeth we sectioned were located next to the “special foramina” in the maxilla and dentary, which represent the sites at which the odontogenic organ, the dental lamina begins forming new teeth at each tooth position [7] (Additional file 4: Figure S3). These teeth were clearly at the earliest stages of dental tissue mineralization, given their proximity to these foramina and their comparable relative size and morphology to the earliest-staged teeth examined by Horner [15]. Teeth at their earliest ontogenetic stages consisted of thin bands of enamel and primary orthodentine (hereafter simply referred to as dentine). These teeth already began forming the attachment tissues at this early stage, as indicated by the presence of typical acellular cementum [16–21] and the first layers of cellular cementum along the surface opposite of the enamel (Fig. 3d, e). Replacement teeth at comparable stages in *Alligator* (Fig. 3f–i; Additional file 5: Figure S4) and theropod dinosaurs (Additional file 6: Figure S5) only consisted of an enamel cap and underlying dentine, and no cementum was present until they neared eruption. The subsequent stage of hadrosaurid tooth ontogeny occurred deep within the jaw and was characterized by extensive deposition of dentine, with the pulp cavity at the tip of the tooth becoming partially closed off (Figs. 3a, c and 4a), and blood vessels being enveloped by the rapid pulpward formation of dentine. This unusual form of dentine development was matched by precocious growth of cellular cementum with Sharpey’s fibers, indicating that the hadrosaurid tooth was already anchored by periodontal ligament (Fig. 4a, d). In other archosaurs and mammals, even nearly erupted teeth have open pulp cavities and lack a ligamentous attachment to the socket (Fig. 3f; Additional file 5: Figure S4, Additional file 6: Figure S5), the latter normally forming during eruption into the oral cavity [11, 16, 22, 23].

The lingual surfaces of unerupted hadrosaurid teeth are partially resorbed by the formation of the subsequent teeth. However, a dentine wall always separates the pulp cavity of an older tooth from the developing tooth underneath (Figs. 3a–e and 4), thus maintaining the vital pulp of the older tooth. In strong contrast, in other vertebrates this process leads to the shedding of old teeth, because the pulp cavity becomes breached by the advancing resorption front of the replacement tooth, which disrupts the vascular supply to the pulp [2]. Prior to tooth eruption in hadrosaurids, the pulp cavity became completely enclosed by rapid deposition of dentine (Figs. 3a, b and 4c–f), first at the occlusal end of the tooth and continuing apically in a zipper-like fashion through the tooth. The advancing walls of dentine converged near the midline of each tooth, creating a line of isolated pockets of the remaining pulp cavity (Figs. 3a–c and 4a). Although it has been commonly thought that the exposed teeth in dental batteries of hadrosaurids were pushed outward from beneath by their younger successors, and that no tooth resorption occurred [2, 7, 24], we found that root resorption was extensive in hadrosaurid teeth (Figs. 1d, f, 3a and 4a, c, e, f; Additional file 4: Figure S3) and instead provided the mechanism through which the teeth became tightly interlocked. The extent of root resorption is demarcated by irregular, jagged surfaces, which are typical of the Howship’s lacunae left by osteoclasts, which form a reversal line (Figs. 1f, g and 4c, f). The presence of extensive root resorption indicates that another mechanism was responsible for continuous tooth eruption in hadrosaurid dental batteries. It is more likely that the periodontal ligament, along with root elongation, served as the agents through which teeth were able to continuously erupt into the oral cavity, similar to the condition in ever-growing teeth in mammals [25]. At the time of eruption into the oral cavity, the teeth of hadrosaurids had nearly completely plugged pulp cavities and thus were probably no longer vital. Sharpey’s fibers were also abundant within the thickened layers of cellular cementum, indicating continued attachment of the tooth to the surrounding socket by periodontal ligament (Fig. 4d). After eruption, each tooth was worn down completely, including the root, instead of being shed (Fig. 4a, e).

### Tooth attachment in the hadrosaurid dental battery

**Fig. 5:**
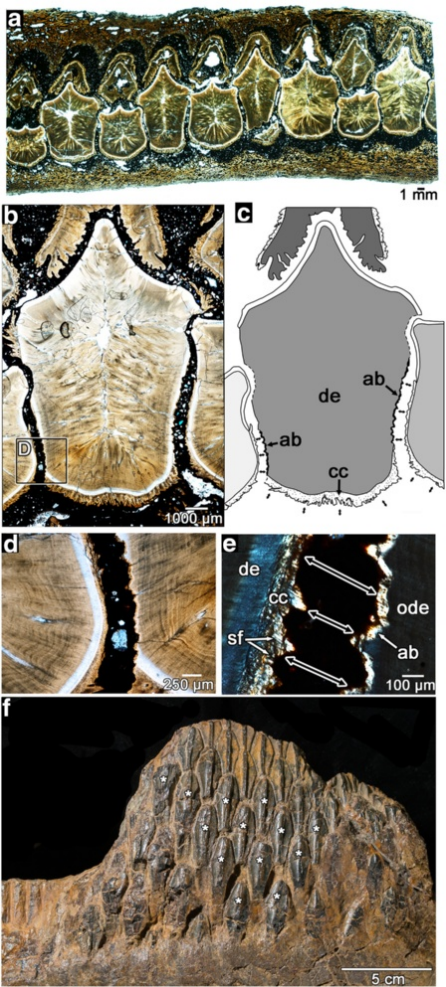
Tooth-to-tooth attachment within the hadrosaurid dental battery. **a** wholeview image of a transverse section through a maxillary battery, near the occlusal surface (labial is towards the top). Note that none of the teeth in the section make hard-tissue contacts to adjacent teeth. **b** closeup image of three neighbouring teeth in the same transverse section of a partial maxilla (ROM 59042). **c** illustration of (**b**) showing positions and orientations of periodontal ligament connections (*black arrows*) between adjacent teeth. Lighter shades of grey indicate younger relative ages for teeth, determined by partial resorption of older neighbouring teeth. Dashed lines indicate tooth resorption. **d** magnified image of (**b**) showing partial resorption of an older tooth (*right*) by a younger tooth (*left*). **e** magnified cross-polarized image of (**d**) showing the development of a thin layer of alveolar bone along the partially resorbed root of the older tooth. The younger tooth developed a periodontal ligament connection (*black arrows*) to the older tooth as indicated by the presence of Sharpey’s fibers. **f** a partial dentary battery of *Edmontosaurus* (ROM 00620) showing several teeth that have moved out of position post-mortem (asterisks), which supports the presence of a soft tissue connection between these teeth in life. ab, alveolar bone; ab/rc, alveolar bone (possible repair cementum); cc, cellular cementum; de, dentine; en, enamel; ode, dentine of older tooth; sf, Sharpey’s fibers

**Fig. 6:**
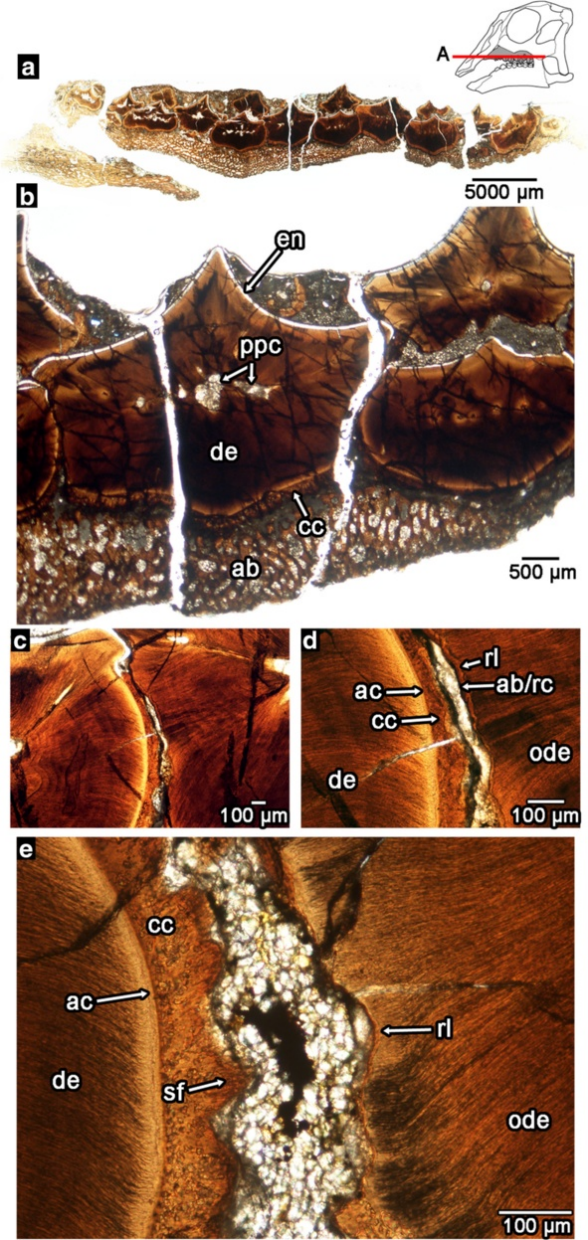
Tooth-to-tooth attachment within an embryonic *Hypacrosaurus* dental battery. **a** wholeview image of a transverse section through a maxillary battery (MOR 559), near the occlusal surface (labial is towards the top). Illustration of *Hypacrosaurus* skull modified from Bailleul et al. [31]. **b** closeup image of maxillary teeth in the same transverse section. **c** closeup image of tooth-to-tooth attachment in MOR 559 (note the lack of contact between the adjacent teeth). **d** closeup image of (**c**) showing partial root resorption and attachment tissues. **e** closeup image of attachment sites of periodontal ligament between two teeth (Sharpey’s fibers). ab, alveolar bone (possible repair cementum between teeth); ac, acellular cementum; cc, cellular cementum; de, dentine; en, enamel; ode, dentine of older tooth; rl, reversal line; sf, Sharpey’s fibers

Since the early discoveries of hadrosaurid dinosaurs, many researchers have presumed that their teeth were coalesced into a massive battery for more efficient grinding [24, 26]. Most recently, Erickson et al. [5] implicated coronal cementum as the tissue that coalesced teeth together. If this interpretation is correct, then hadrosaurids would represent the first dinosaur taxon to exhibit ankylosis, or fusion of teeth by hard tissue; however, we found no evidence of any hard tissue bridging the gaps between teeth at any ontogenetic stage (Figs. 4, 5 and 6; Additional file 2: Figure S1, Additional file 4: Figure S3), and only found sediment infilling between all of the teeth within the batteries. This infilling is barely noticeable along the surfaces of specimens and can only be confirmed in thin sections of intact, in situ batteries, all of which show this phenomenon. This also occurs in fossil mammals, crocodilians, and even other dinosaurs, because their teeth were suspended by the soft tissue of the periodontal ligament in life, which decayed after death, and was replaced by sediment or diagenetic minerals [9, 13, 16, 27] (Figs. 5 and 6, Additional file 2: Figure S1). All of the teeth within the battery were suspended by periodontal ligaments to thin layers of alveolar bone that lined the labial and lingual walls of the jawbone. This form of tooth attachment, called a gomphosis, is found in many stem and crown amniotes [16, 20, 28], including other archosaurs [9, 10, 22] (Additional file 2: Figure S1). Within a single stack of maxillary teeth (a tooth family, sensu [2]), the younger teeth made no hard or soft tissue connections to the older teeth above them, because of the presence of an intervening layer of enamel from the younger teeth (Fig. 4). Conversely, the mirrored arrangement of the enamel in the dentary teeth meant that ligamentous connections between successive generations of teeth within a vertical stack could occur, but not to the lingual wall of the dentary (Additional file 7: Figure S6). Surprisingly, new teeth formed thin ligamentous attachments against the partially resorbed dentine bases of older, neighbouring teeth (Figs. 5a–e and 6) in longitudinal section. Sharpey’s fibers extend from the cellular cementum of younger teeth to a thin layer of avascular, bone-like tissue covering the resorbed dentine of older teeth (Figs. 5 and 6). This bone tissue either represents a thin layer of alveolar bone or repair cementum [29, 30] that formed after partial root resorption and anchored the collagen fiber bundles of the adjoining periodontal ligament. Sharpey’s fibers are visible under cross-polarized light, marking the former positions of the ligament fibers between each tooth (Fig. 5e). Each tooth was therefore suspended by periodontal ligament to the walls of the jaw and to neighbouring teeth. We observed these patterns of tooth attachment in hadrosaurid dental batteries of large and embryonic individuals (Figs. 5 and 6, see [31, 32] for assessment of studied material as embryonic and nestlings).

## Discussion

### The evolution of the hadrosaurid tooth

Our ontogenetic perspective reveals that the histological complexity and the elaborate life cycle of hadrosaurid teeth did not involve the evolution of novel tissues, as previously suggested [5]. Hadrosaurid teeth are composed of homologous tissues to those in other archosaurs, and other amniotes in general [9, 13, 16–19, 23, 27, 28, 33] (Additional file 2: Figure S1). Hadrosaurid teeth are unique among reptilian dentitions in that the arrangements of homologous tissues forming each tooth have been radically altered from the ancestral dinosaurian condition (Fig. 2). Whereas the enamel of the typical reptilian tooth crown forms a cap over the apex of a tooth (thus forming the anatomical crown [11]), the enamel of hadrosaurid teeth is restricted to one side of the tooth [2]. As a result, the junction between cementum, typically a root tissue [12], and the crown tissue enamel has shifted to the apex of the hadrosaurid tooth (Fig. 2h). This shift in the cemento-enamel junction means that each tooth begins forming enamel and cementum simultaneously in the early stages of dental development. This also means that the enamel-covered face of the tooth does not contribute to tooth attachment, which is anatomically more similar to the condition in mammalian ever-growing incisors [12, 34] than to the complex grinding teeth of modern horses [35, 36]. From evolutionary and developmental perspectives, the asymmetrical deposition of enamel in hadrosaurid teeth should not be confused with the evolution and development of coronal cementum in mammals. Hadrosaurids did not independently evolve coronal cementum (contra [5, 8]), because coronal cementum occurs as an external covering of the enamel and is the result of a different developmental process [16, 37] (Additional file 8: Figure S7). The cellular cementum in hadrosaurid teeth never overlaps the enamel as it does in many ungulate mammals. Instead, typical root cementum extends to the tooth apex in hadrosaurids and the cementum becomes incorporated into the occlusal surface through a combination of its unique orientation along the tooth, constant eruption, and wear of the whole tooth (Fig. 4).

### Heterochrony and the evolution of the hadrosaurid dental battery

Our comparison of hadrosaurid dental ontogeny to that of other amniotes also provides clear evidence of an accelerated rate of dentine and cementum formation in hadrosaurids. We invoke heterochrony, namely the earlier onset and increased rate of tissue formation as the evolutionary mechanism through which hadrosaurids were able to evolve complex dental batteries (Fig. 3). The vascular nature of the dentine and cementum in hadrosaurid teeth is the hallmark of an accelerated rate of tissue formation that entombed blood vessels of the pulp and the periodontium, not the evolution of new types of tissues as previously suggested [5]. When compared to other amniotes, hadrosaurid teeth were not only forming more rapidly [38], but also matured earlier. Dentine was deposited so precociously and extensively that the soft tissue within the pulp cavity was completely obliterated by the time the tooth erupted (Figs. 2, 3 and 4; Additional file 4: Figure S3). The infilling of the pulp with dentine created not only a wear surface for grinding plant material [5], but also served the more critical function of preventing a breach of the pulp cavity. Hadrosaurid teeth were ground down over the course of their use [2, 5, 7], and a breached pulp cavity, caused by attrition or root resorption, could normally lead to infection and severe pain, given the density of blood vessels and nerves in the dental pulp [11]. Cementum in most amniotes is typically avascular [11, 16], but a similar condition occurs in modern horses, where the entombed vasculature of the coronal cementum recedes as the tooth erupts into the oral cavity [35]. In addition, the rapid closure of the pulp cavity would have initially prevented tooth replacement, but once completed, the tooth would have also been non-vital, given the lack of nourishment to the living tissues of the tooth. Filling the pulp cavity with dentine thus provided a remarkable solution to the challenge of tooth wear: during their ontogeny, hadrosaurids were able to grind down an entire tooth because it was completely infilled with dentine while still retaining a connection to its neighbouring teeth, thus allowing dead teeth to remain functional as grinding surfaces. Hadrosaurid teeth were never shed as they are in other vertebrates, but were worn away completely, allowing hadrosaurids to grind away at multiple teeth at once in a single tooth position [1, 39]. This functionalization of the tooth root into the grinding surface was permitted by the connections between teeth within the dental battery, and represents a true evolutionary novelty among vertebrates.

Accelerated cementum formation indicates extensive attachment of hadrosaurid teeth to their surroundings, but in a unique fashion. Previous suggestions that cementum in hadrosaurids served to fuse the teeth together into a single grinding pavement [5], are incorrect. In thin section, none of the teeth within a battery contact each other (Figs. 1, 4, 5 and 6; Additional file 4: Figure S3, Additional file 7: Figure S6). Cementum was deposited comparatively early in dental ontogeny, at the same time as enamel and dentine (Fig. 3), anchoring the teeth by ligament to the alveoli. We find further support for a ligamentous attachment of hadrosaurid teeth based on taphonomy. Fossil and modern skeletonized jaws of mammals and crocodilians are typically edentulous due to post-mortem tooth loss, which is rare in taxa that have teeth fused to the jaws [16, 40]. This phenomenon occurs because the soft tissues of the periodontal ligaments decay after death, severing the connection between the teeth and the alveoli [16]. The mode of preservation of hadrosaurid dental batteries is inconsistent with the model that hadrosaurid teeth were fused together by hard tissue. As we and others [2] have shown, hadrosaurid teeth were not shed, but ground down completely, indicating that isolated hadrosaurid teeth are actually the remains of dissociated dental batteries. Many Late Cretaceous vertebrate microsites are replete with isolated hadrosaurid teeth [41–43] and many dental batteries preserve clusters of teeth that have become dissociated from the rest of the battery [39, 44] (Fig. 5f), which further support a soft tissue attachment between individual teeth. This re-interpretation of hadrosaurid tooth attachment not only more accurately explains the mechanisms underlying hadrosaurid dental taphonomy, but also has important implications for the evolution and function of the hadrosaurid dentition.

Whereas all dinosaurs studied to date possessed a ligamentous tooth attachment system [9, 45, 46], the hadrosaurid dental battery is unique in vertebrate evolution because the entire assembly, as well as individual teeth, were suspended within the jaw by ligaments. Even more surprisingly, individual teeth were suspended to their neighbors within the battery by periodontal ligaments from the onset of tooth development, thus allowing the whole battery to continuously erupt and respond to the compressive forces of chewing as a complex unit. The periodontal ligament permits teeth to erupt into the oral cavity in mammals with continuously-erupting teeth [25, 47, 48] and hadrosaurids exhibit an analogous condition using the same dental tissues. The unique form of tooth-to-tooth attachment in hadrosaurids also provides clear evidence of the modular nature of developing teeth [49, 50], because each developing tooth bud in hadrosaurids formed its own periodontal tissues to which it was attached by a ligament. This would have provided a tremendous mechanical advantage to the dental battery, probably even greater than in mammals. In mammals, the periodontal ligament serves as a shock absorber to dissipate the forces of dental occlusion [11] between individual teeth aligned in a single row mesiodistally. Given the grinding motions that hadrosaurs employed to consume plant material [5], a grinding battery possessing several hundred small teeth that were individually suspended by ligaments would have been extremely advantageous. The sheer number and small sizes of these interconnected teeth (much smaller than in ornithopods that did not possess dental batteries [6]), and their sophisticated ontogeny demonstrate that these dinosaurs evolved a more complex dental system than herbivorous mammals. The differences between hadrosaurid dental batteries and the grinding teeth of mammalian herbivores show convergent solutions to the problem of constant tooth wear. The major differences between ungulate mammals and hadrosaurids relate to the fact that mammals have lost continuous tooth replacement and thus individual teeth are extensively modified for efficient grinding, whereas hadrosaurid dental batteries take advantage of reptilian polyphyodonty.

We also found the same tissue arrangements and ligamentous tooth attachments in nestling (MOR 548) and embryonic (MOR 559) hadrosaurid jaws (Fig. 6). Each of these specimens shows the development of multiple generations of teeth at a single locus, erupted teeth with plugged pulp cavities, partial root resorption of neighbouring teeth, and clear evidence of ligamentous tooth-to-tooth attachments across tooth positions (Fig. 6). These data suggest that the teeth of embryonic and neonatal hadrosaurids were similarly advanced in their degree of tooth tissue formation compared to adults. Late-stage embryos of modern crocodilians also develop, and even replace, multiple generations of teeth prior to hatching [51–53], but the presence of multiple generations of interconnected teeth in an embryonic and neonatal hadrosaurid suggests that the formation of a dental battery began *in ovo* and may have been functional immediately after hatching.

Dental batteries have evolved independently in ornithischian (Neoceratopsia, Hadrosauridae) and saurischian (Rebbachisauridae) dinosaurs [2, 54–56], however, the present study is the first to examine dental battery development and evolution at the histological level. Therefore, the uniqueness of hadrosaurid dental ontogeny and histology depends on further comparisons with the dentitions of neoceratopsians and rebbachisaurid sauropods. One key prediction that can be made from this work is that future studies in the aforementioned taxa will uncover adaptations to avoiding typical amniote tooth replacement either by accelerated tooth development (as in hadrosaurids) or by some other means of spatially separating older teeth from the dental lamina [57, 58]. A brief survey of descriptions of sauropod dentitions shows that they consist of numerous generations of replacement teeth that are spatially separated from one another and gradually migrate to their functional positions through ontogeny [54, 55, 59]. By comparison, the stacked teeth of the neoceratopsian dental battery consist of multiple generations of teeth that are closely packed, apparently more so than in hadrosaurids [56]. Each developing neoceratopsian tooth appears to be nested within the pulp cavity of its predecessor [56], a phenomenon that does not occur in hadrosaurid teeth. These brief comparisons suggest that studying dental battery evolution in dinosaurs will reveal different ways in which dental ontogeny has been modified to produce novel dental systems.

The evolutionary and ontogenetic model we have proposed here may also help explain other aspects of hadrosaurid dental histology. Although the enamel of hadrosaurid teeth is clearly homologous to that of other amniotes, the enamel surfaces of hadrosaurid teeth possess a rough surface micromorphology consisting of microscopic enamel globules [60–62]. Sander [61] hypothesized that the globular surface texture of hadrosaurid teeth, which results in a dull luster to the enamel surface (Fig. 1c), may have evolved as a peculiarity of dental battery development, given that it is only found in hadrosaurid and neoceratopsian teeth. Sander [61] proposed that the micrometer-scale enamel globules along the surfaces of hadrosaurid and neoceratopsian teeth may be the byproduct of crowding of the inner dental epithelium during amelogenesis, but this was probably not the case, given that hadrosaurid teeth resorbed significant portions of adjacent teeth in order to accommodate their development (Figs. 4c and 5). If this feature is not an adaptation to extensive tooth wear, then another plausible explanation is that it is related to the relatively rapid deposition of enamel, similar to the rapid rates of dentine and cementum deposition. However, further detailed examinations of enamel surface microstructure at several ontogenetic stages using Scanning Electron Microscopy are needed in order to test this hypothesis, as this surface morphology would be predicted to be present at all ontogenetic stages of enamel formation.

## Conclusions

The first ornithopod taxa to retain more than one generation of replacement teeth were basal hadrosauroids that appeared in the Early Cretaceous, with the highly integrated dental batteries evolving in tandem with further tooth proliferation and miniaturization in Late Cretaceous Hadrosauridae [1, 39]. Our findings show that the novelty of the hadrosaurid dental system lies in how ancestral reptilian dental tissues were used in the interaction between individual teeth and between families of teeth. Each tooth is analogous to a single scale on medieval armor, or a single denticle in shark skin, where each scale or denticle is a rigid structure, whereas the interconnecting material provides flexibility [63]. Despite the structural complexity of their batteries, dental development in hadrosaurids was constrained by the same processes that govern tooth formation in all other amniotes, given that each tooth is composed of homologous tissues to those in stem and crown Amniota [16, 49, 50]. We not only attribute the differences between hadrosaurid dental development and other amniotes to a shift in the orientation of typical crown and root tissues, but also to heterochronic acceleration [64], which allowed hadrosaurids to halt tooth replacement, co-opt the tooth roots into the grinding surface, and maintain multiple generations of functional teeth at the same locus [7, 39, 65]. Heterochrony in dental development was therefore a key evolutionary event that promoted the rapid diversification of hadrosaurid dinosaurs, the dominant herbivores in many Late Cretaceous communities [1, 39].

## Abbreviations

CMN, Canadian Museum of Nature, Ottawa, Ontario, Canada; MOR, Museum of the Rockies, Bozeman, Montana, U. S. A.; ROM, Royal Ontario Museum, Toronto, Ontario, Canada; UMNH, Natural History Museum of Utah, Salt Lake City, Utah, U. S. A.; USNM, National Museum of Natural History, Washington, D. C

## Additional files

Additional file 1: Table S1. Thin sections examined in this study. (DOCX 69 kb) Additional file 2: Figure S1. Comparisons of amniote periodontal tissues. (A) closeup image of the periodontal tissues in a hadrosaurid tooth in transverse section (ROM 59042). (B) closeup image of the periodontal tissues in a hadrosaurid tooth in cross-polarized light to show orientations of Sharpery’s fibers of the periodontal ligament. (C) closeup image of the periodontal tissues in a tyrannosaurid dinosaur tooth in transverse section (CMN 2225). (D) closeup image of same tooth under cross-polarized light. (E) closeup image of the periodontal tissues in a large, subfossil *Alligator mississippiensis* tooth in longitudinal section (ROM 21496). (F) closeup image of periodontal tissues in transverse section of same specimen in cross-polarized light. (G) closeup image of periodontal tissues in the fossil mammal *Hyopsodus* (USNM 595273). (H) closeup image of periodontal tissues in same specimen under cross-polarized light. Abbreviations: ab, alveolar bone; bo, bone of the jaw; ac, acellular cementum; cc, cellular cementum; de, dentine; en, enamel; ode, dentine of older tooth; ps, periodontal space; sf, Sharpey’s fibers. (TIF 3235 kb) Additional file 3: Figure S2. Labeled model of the hadrosaurid maxillary dental battery (illustration by Danielle Dufault). Abbreviations: ab, alveolar bone; ab/rc, alveolar bone (possible repair cementum); ac, acellular cementum; bv, blood vessels; cc, cellular cementum; de, dentine; do, denteon; en, enamel; jb, bone of the jaw; Li, lingual; lp, lingual plate; Me, mesial; spf, “special foramen”; pdl, periodontal ligament; pc, pulp cavity; ppc, plugged pulp cavity; rp, resorption pit. (TIF 6427 kb) Additional file 4: Figure S3. Histological sections through the maxillae of two hadrosaurid dental batteries (ROM 00696, ROM 59042). (A) coronal section through a maxillary dental battery (ROM 00696) showing three generations of erupted teeth. (B) coronal section through the same maxillary dental battery showing three generations of unerupted teeth. These two sections were used to create the ontogenetic sequence of hadrosaur teeth presented in Fig. 3. (C) longitudinal section through the maxilla of ROM 59042 showing the lack of fusion of any of the teeth within the maxillary battery. (TIF 9243 kb) Additional file 5: Figure S4.
*Alligator* tooth development viewed in serial sections of a 40-day old *Alligator* (ROM R6252). (A), section through a functional tooth and a newly developing replacement tooth. (B) section through a functional tooth with a larger replacement tooth that has invaded the pulp cavity. (C) section through a tooth position in which the functional tooth has been shed and the new tooth has not yet erupted into the oral cavity. (D) section in which a newly erupted functional tooth has formed a ligamentous connection to the alveolus, with a new tooth beginning to form lingually. These sections were used to reconstruct dental ontogeny in *Alligator* in Fig. 3. Abbreviations: ac, acellular cementum; cc, cellular cementum; de, dentine; en, enamel; pdl, periodontal ligament. (TIF 4685 kb) Additional file 6: Figure S5. Relative timing of dental development in a hadrosaurid and the theropod dinosaur *Allosaurus*. (A) tooth development sequence in a hadrosaurid (ROM 00696). (B) closeup image of the plugged pulp cavity of an erupting hadrosaurid tooth. (C) closeup image of the plugged pulp cavity of an unerupted hadrosaurid tooth. (D) closeup image of the root tissues of an unerupted hadrosaurid tooth. (E) closeup image of the root tissues of a newly formed hadrosaurid tooth. (F) pre-eruptive tooth development sequence in *Allosaurus* (UMNH 23781). (G) closeup image of the tooth tissues in a nearly erupted *Allosaurus* tooth. (H) closeup image of the tooth tissues in a newly formed tooth. Abbrevations: ac, acellular cementum; cc, cellular cementum; de, dentine; do, denteon; en, enamel; pc, pulp cavity; ppc, plugged pulp cavity. (TIF 4621 kb) Additional file 7: Figure S6. Tooth attachment in the hadrosaurid dentary dental battery. (A) overview image of a thin section through a dentary of the hadrosaudid *Prosaurolophus* (ROM 03500). (B) closeup image of the lingual surface of the dental battery and the overlying lingual plate of bone. (C) closeup image of the labial surface of the dental battery and the labial wall of alveolar bone. (D) closeup image of the periodontal ligament attachment between successive generations of teeth within the dentary dental battery. (E) same image as in D, but in cross-polarized light, showing orientations of Sharpey’s fibers of the periodontal ligament (black arrows). For all images, lingual is to the left. Abbreviations: ab, alveolar bone, cc, cellular cementum, de, dentine; en, enamel; jb, bone of the jaw; lp, lingual plate; ode, dentine of older tooth; ps, periodontal space. (TIF 2247 kb) Additional file 8: Figure S7. Histology of the dentine and coronal cementum in a horse. (A) illustration of the major tissues in a horse tooth in longitudinal view. (B) overview image of a transverse section through a horse tooth (ROM 33036). (C) closeup image of the coronal cementum of a horse, which overlies the enamel of the tooth crown. (D) closeup image of the infundibular cementum in a horse showing abundance of cementocytes and occasional vascular spaces, which are surrounded by cementeons. (E) closeup image of coronal cementum under cross-polarized light, showing orientations of Sharpey’s fibers of the periodontal ligament. (F) closeup image of periodontal tissues of a horse. Abbrevations: ab, alveolar bone; cc, coronal cellular cementum; de, dentine; en, enamel; pc, pulp cavity; pd, primary dentine; rl, reversal line; sd, secondary dentine; sf, Sharpey’s fibers. (TIF 8893 kb)
