# Supplementary material for: Ontogeny reveals function and evolution of the hadrosaurid dinosaur dental battery
Source: BMC Evol Biol. 2016 Jul 28;16:152. doi: 10.1186/s12862-016-0721-1 (PMC4964017; doi:10.1186/s12862-016-0721-1)
Supplement: Additional file 1: Table S1. — Thin sections examined in this study. (DOCX 69 kb) [file 12862_2016_721_MOESM1_ESM.docx]

**Table 1. Specimens examined and sectioned in this study.**

| **Taxon** | **Specimen number** | **Thin section description** |
| --- | --- | --- |
| Hadrosauridae indet. | ROM 00696 | Three coronal sections through a partial maxilla |
| *Prosaurolophus* | ROM 03500 | Two coronal sections through partial dentary |
| Hadrosauridae indet. | ROM 59042 | Two longitudinal sections through a partial maxilla |
| *Hypacrosaurus stebengeri* | MOR 559 | Seven longitudinal sections through an embryonic maxilla |
| *Hypacrosaurus stebengeri* | MOR 548 | Longitudinal section through a hatchling dentary |
| Hadrosauridae indet. | ROM 58630 | Two longitudinal and two transverse sections through two isolated teeth |
| Hadrosauridae indet. | ROM 59041 | Transverse section through isolated tooth |
| cf. *Gorgosaurus libratus* | CMN 2225 | Two longitudinal sections through a partial dentary |
| *Allosaurus fragilis* | UMNH 23781 | Coronal section through a partial dentary |
| *Alligator mississippiensis* | ROM R6252 | Four coronal sections through complete dentary of hatchling |
| *Alligator mississippiensis* | ROM 21496 | Two coronal and two longitudinal sections through a partial dentary |
| *Hyopsodus sp.* | USNM 595273 | Two coronal and two longitudinal sections through a partial dentary |
| *Equus sp.* | ROM 33036 | Coronal and longitudinal sections through a partial dentary |
