# Supplementary figures and images for: Ontogeny reveals function and evolution of the hadrosaurid dinosaur dental battery
Source: BMC Evol Biol. 2016 Jul 28;16:152. doi: 10.1186/s12862-016-0721-1 (PMC4964017; doi:10.1186/s12862-016-0721-1)

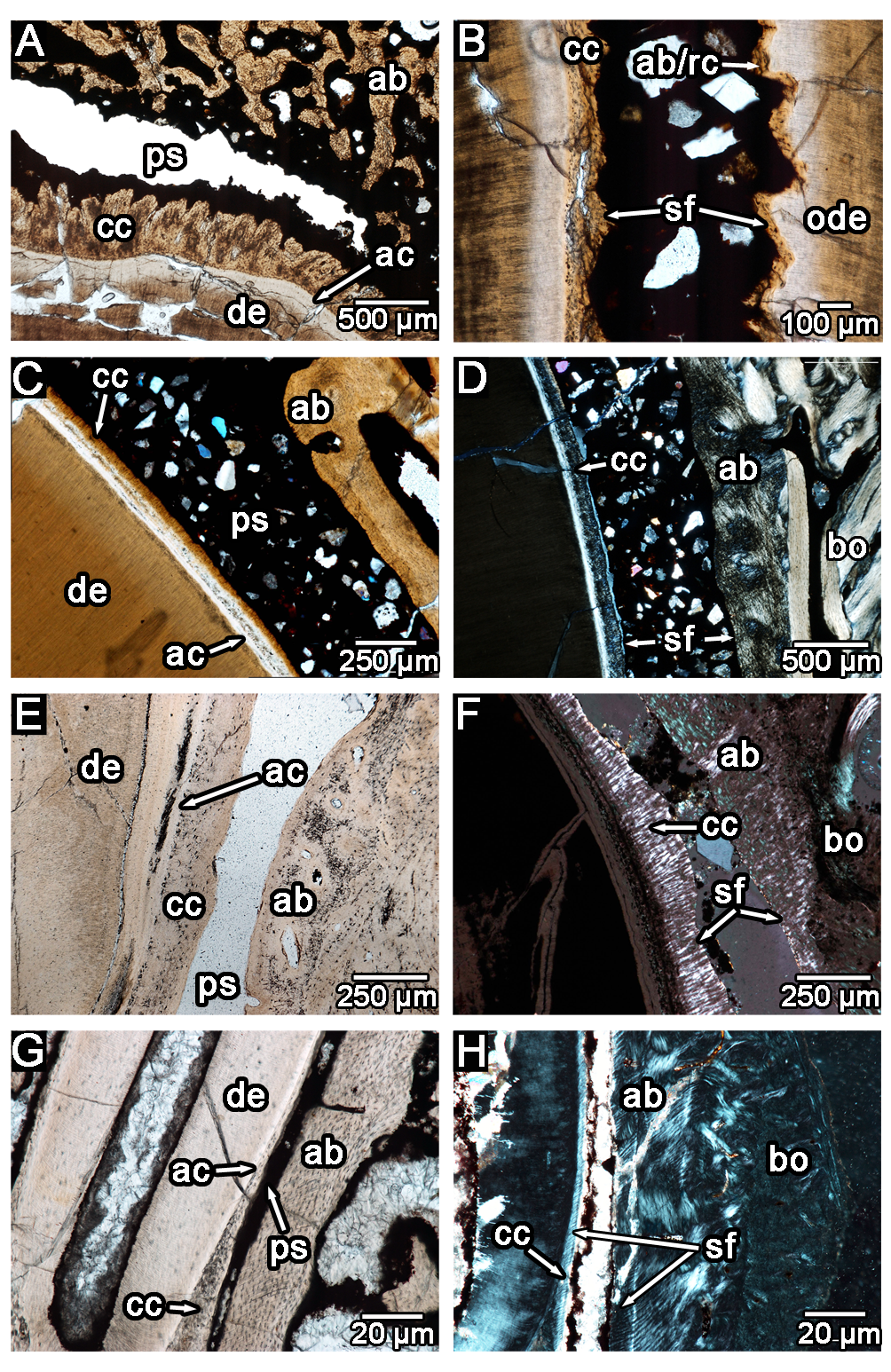

Supplement: Additional file 2: Figure S1. — Comparisons of amniote periodontal tissues. (A) closeup image of the periodontal tissues in a hadrosaurid tooth in transverse section (ROM 59042). (B) closeup image of the periodontal tissues in a hadrosaurid tooth in cross-polarized light to show orientations of Sharpery’s fibers of the periodontal ligament. (C) closeup image of the periodontal tissues in a tyrannosaurid dinosaur tooth in transverse section (CMN 2225). (D) closeup image of same tooth under cross-polarized light. (E) closeup image of the periodontal tissues in a large, subfossil Alligator mississippiensis tooth in longitudinal section (ROM 21496). (F) closeup image of periodontal tissues in transverse section of same specimen in cross-polarized light. (G) closeup image of periodontal tissues in the fossil mammal Hyopsodus (USNM 595273). (H) closeup image of periodontal tissues in same specimen under cross-polarized light. Abbreviations: ab, alveolar bone; bo, bone of the jaw; ac, acellular cementum; cc, cellular cementum; de, dentine; en, enamel; ode, dentine of older tooth; ps, periodontal space; sf, Sharpey’s fibers. (TIF 3235 kb) [file 12862_2016_721_MOESM2_ESM.tif]

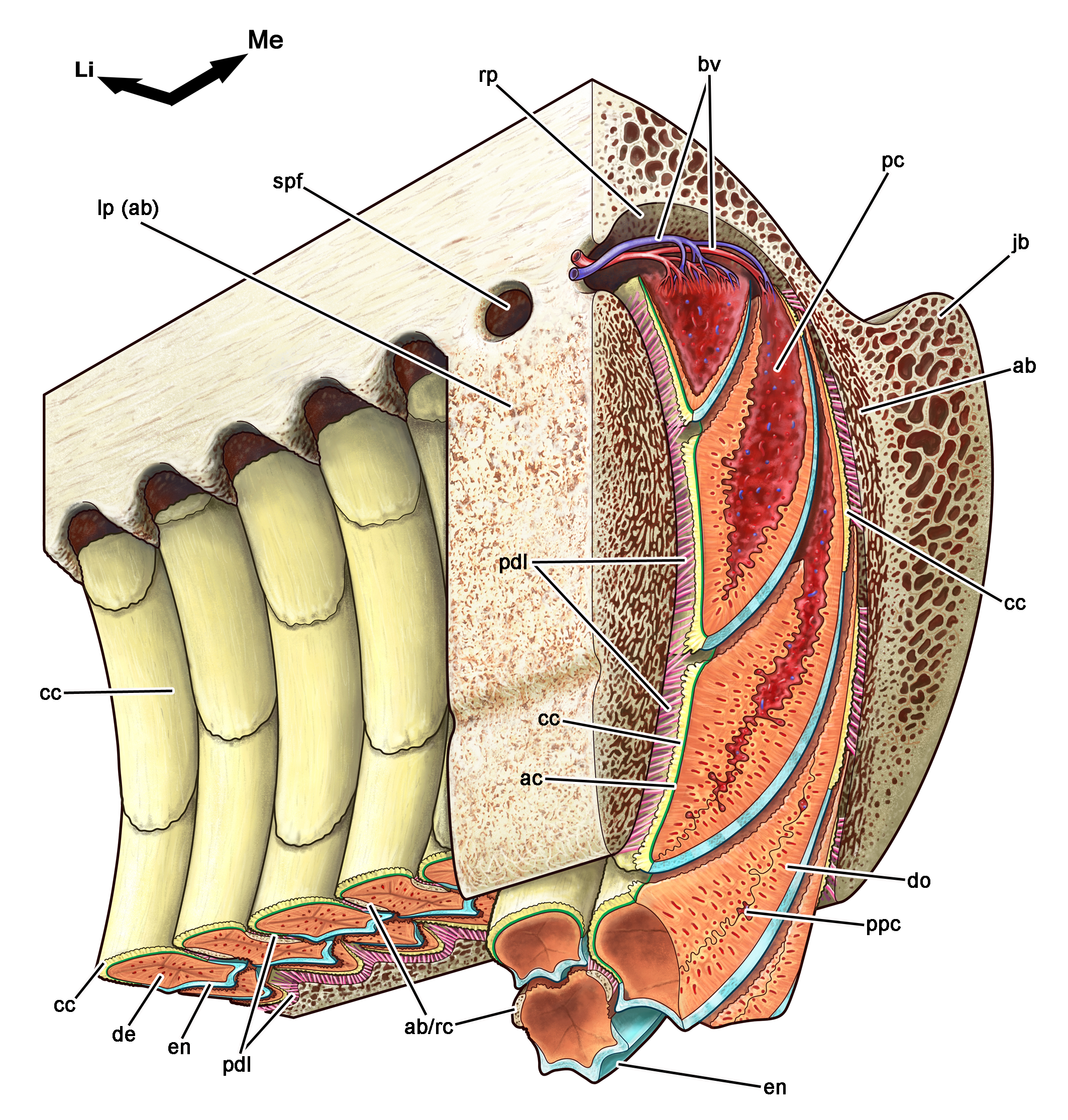

Supplement: Additional file 3: Figure S2. — Labeled model of the hadrosaurid maxillary dental battery (illustration by Danielle Dufault). Abbreviations: ab, alveolar bone; ab/rc, alveolar bone (possible repair cementum); ac, acellular cementum; bv, blood vessels; cc, cellular cementum; de, dentine; do, denteon; en, enamel; jb, bone of the jaw; Li, lingual; lp, lingual plate; Me, mesial; spf, “special foramen”; pdl, periodontal ligament; pc, pulp cavity; ppc, plugged pulp cavity; rp, resorption pit. (TIF 6427 kb) [file 12862_2016_721_MOESM3_ESM.tif]

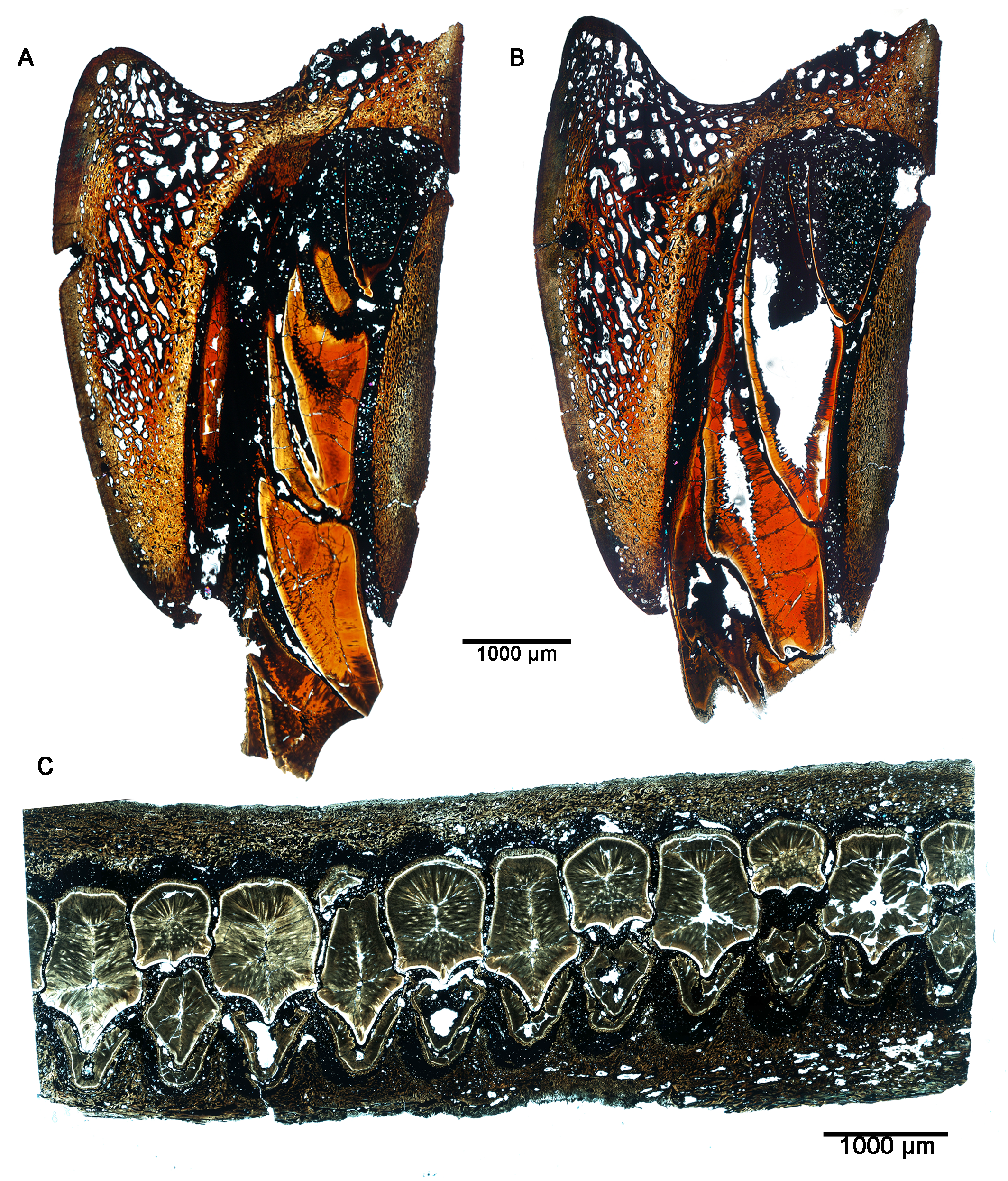

Supplement: Additional file 4: Figure S3. — Histological sections through the maxillae of two hadrosaurid dental batteries (ROM 00696, ROM 59042). (A) coronal section through a maxillary dental battery (ROM 00696) showing three generations of erupted teeth. (B) coronal section through the same maxillary dental battery showing three generations of unerupted teeth. These two sections were used to create the ontogenetic sequence of hadrosaur teeth presented in Fig. 3. (C) longitudinal section through the maxilla of ROM 59042 showing the lack of fusion of any of the teeth within the maxillary battery. (TIF 9243 kb) [file 12862_2016_721_MOESM4_ESM.tif]

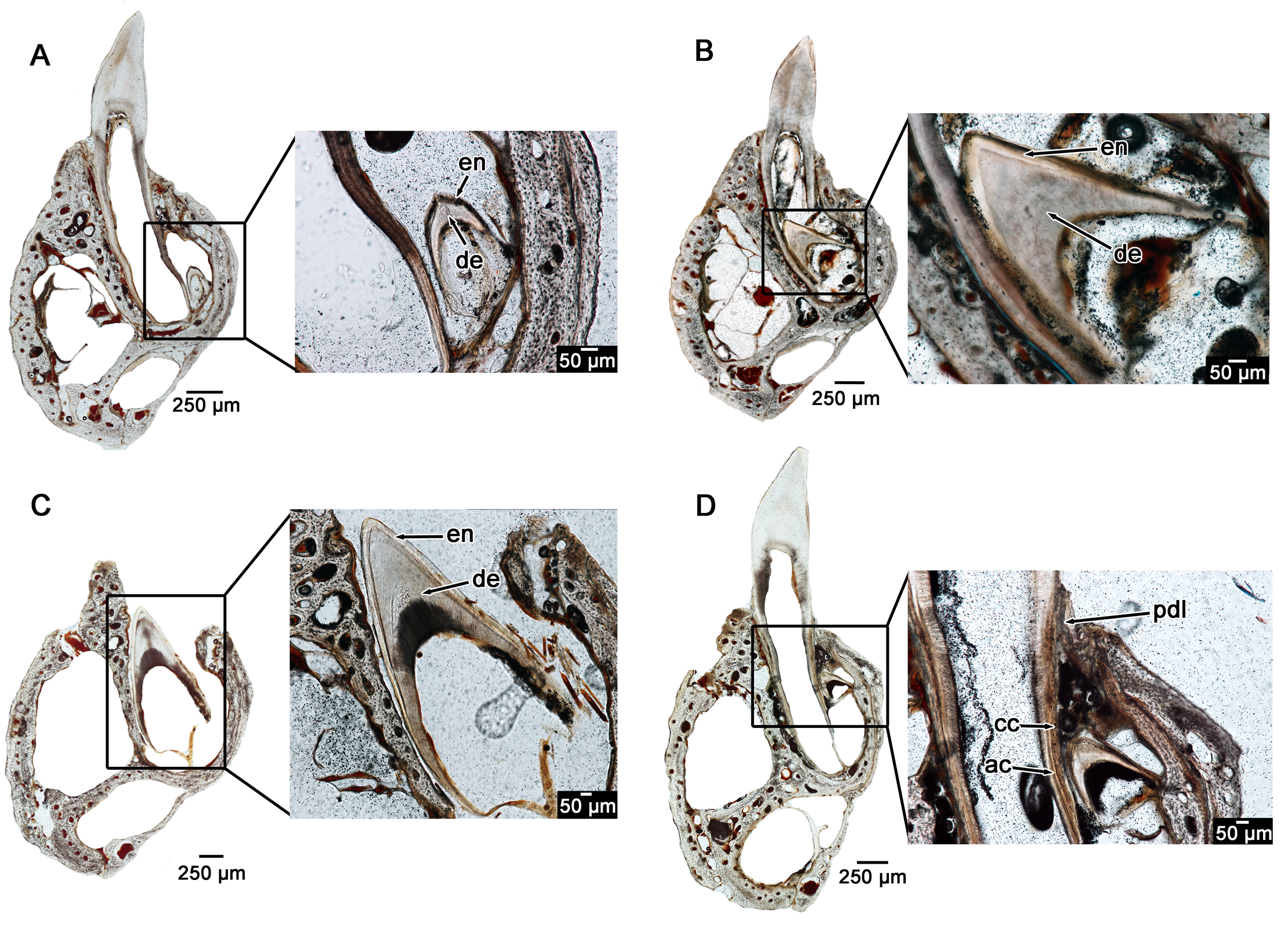

Supplement: Additional file 5: Figure S4. — Alligator tooth development viewed in serial sections of a 40-day old Alligator (ROM R6252). (A), section through a functional tooth and a newly developing replacement tooth. (B) section through a functional tooth with a larger replacement tooth that has invaded the pulp cavity. (C) section through a tooth position in which the functional tooth has been shed and the new tooth has not yet erupted into the oral cavity. (D) section in which a newly erupted functional tooth has formed a ligamentous connection to the alveolus, with a new tooth beginning to form lingually. These sections were used to reconstruct dental ontogeny in Alligator in Fig. 3. Abbreviations: ac, acellular cementum; cc, cellular cementum; de, dentine; en, enamel; pdl, periodontal ligament. (TIF 4685 kb) [file 12862_2016_721_MOESM5_ESM.tif]

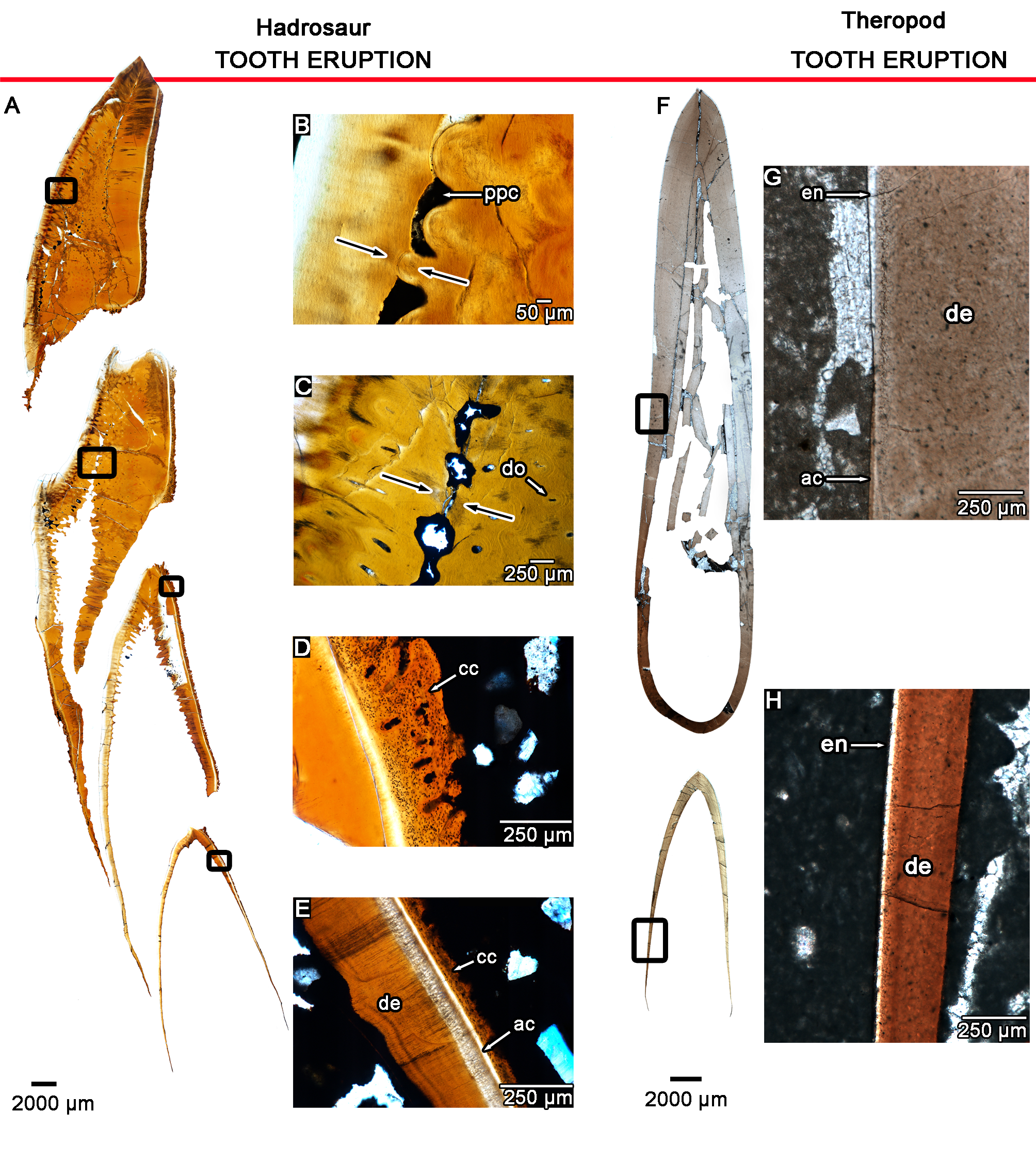

Supplement: Additional file 6: Figure S5. — Relative timing of dental development in a hadrosaurid and the theropod dinosaur Allosaurus. (A) tooth development sequence in a hadrosaurid (ROM 00696). (B) closeup image of the plugged pulp cavity of an erupting hadrosaurid tooth. (C) closeup image of the plugged pulp cavity of an unerupted hadrosaurid tooth. (D) closeup image of the root tissues of an unerupted hadrosaurid tooth. (E) closeup image of the root tissues of a newly formed hadrosaurid tooth. (F) pre-eruptive tooth development sequence in Allosaurus (UMNH 23781). (G) closeup image of the tooth tissues in a nearly erupted Allosaurus tooth. (H) closeup image of the tooth tissues in a newly formed tooth. Abbrevations: ac, acellular cementum; cc, cellular cementum; de, dentine; do, denteon; en, enamel; pc, pulp cavity; ppc, plugged pulp cavity. (TIF 4621 kb) [file 12862_2016_721_MOESM6_ESM.tif]

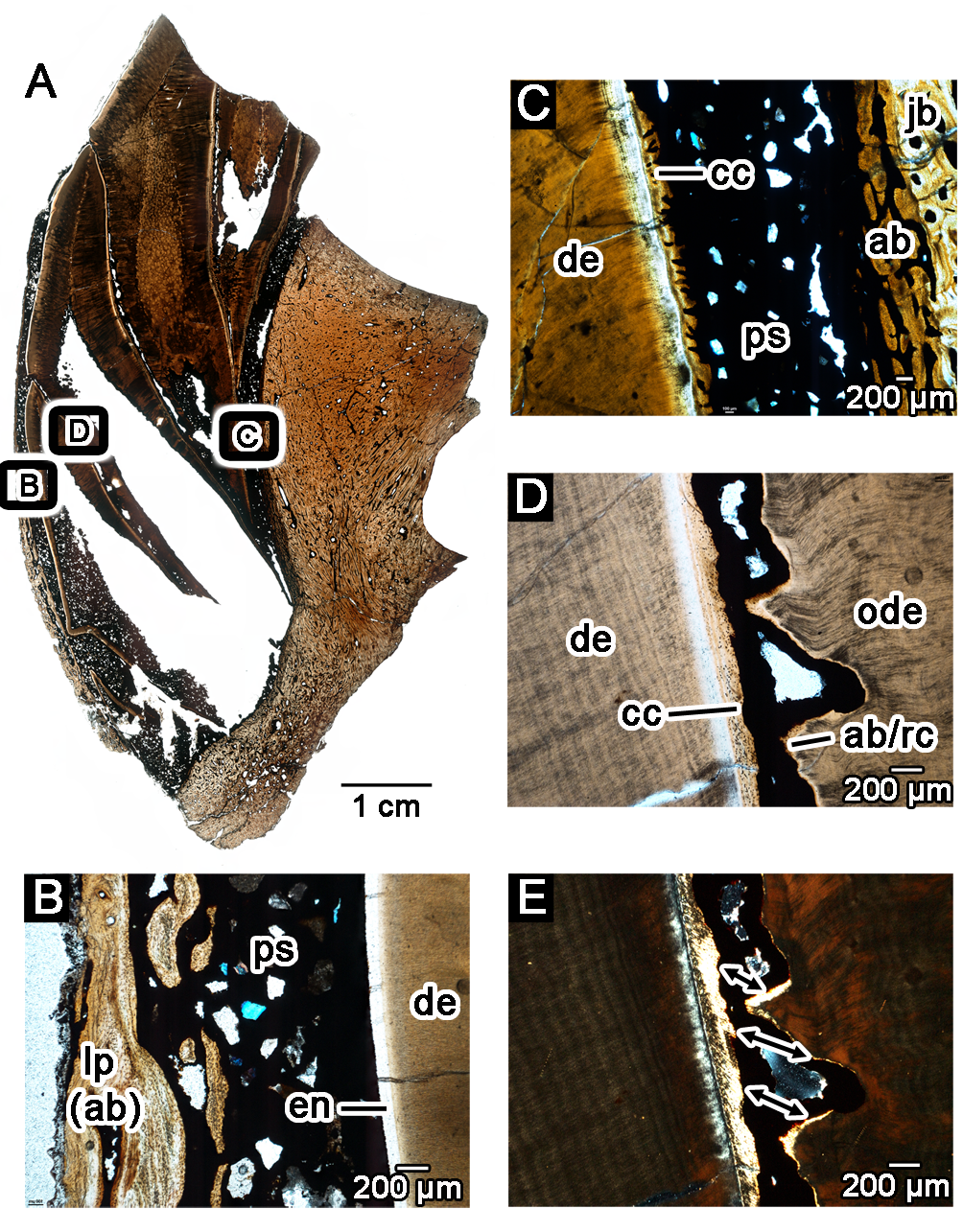

Supplement: Additional file 7: Figure S6. — Tooth attachment in the hadrosaurid dentary dental battery. (A) overview image of a thin section through a dentary of the hadrosaudid Prosaurolophus (ROM 03500). (B) closeup image of the lingual surface of the dental battery and the overlying lingual plate of bone. (C) closeup image of the labial surface of the dental battery and the labial wall of alveolar bone. (D) closeup image of the periodontal ligament attachment between successive generations of teeth within the dentary dental battery. (E) same image as in D, but in cross-polarized light, showing orientations of Sharpey’s fibers of the periodontal ligament (black arrows). For all images, lingual is to the left. Abbreviations: ab, alveolar bone, cc, cellular cementum, de, dentine; en, enamel; jb, bone of the jaw; lp, lingual plate; ode, dentine of older tooth; ps, periodontal space. (TIF 2247 kb) [file 12862_2016_721_MOESM7_ESM.tif]

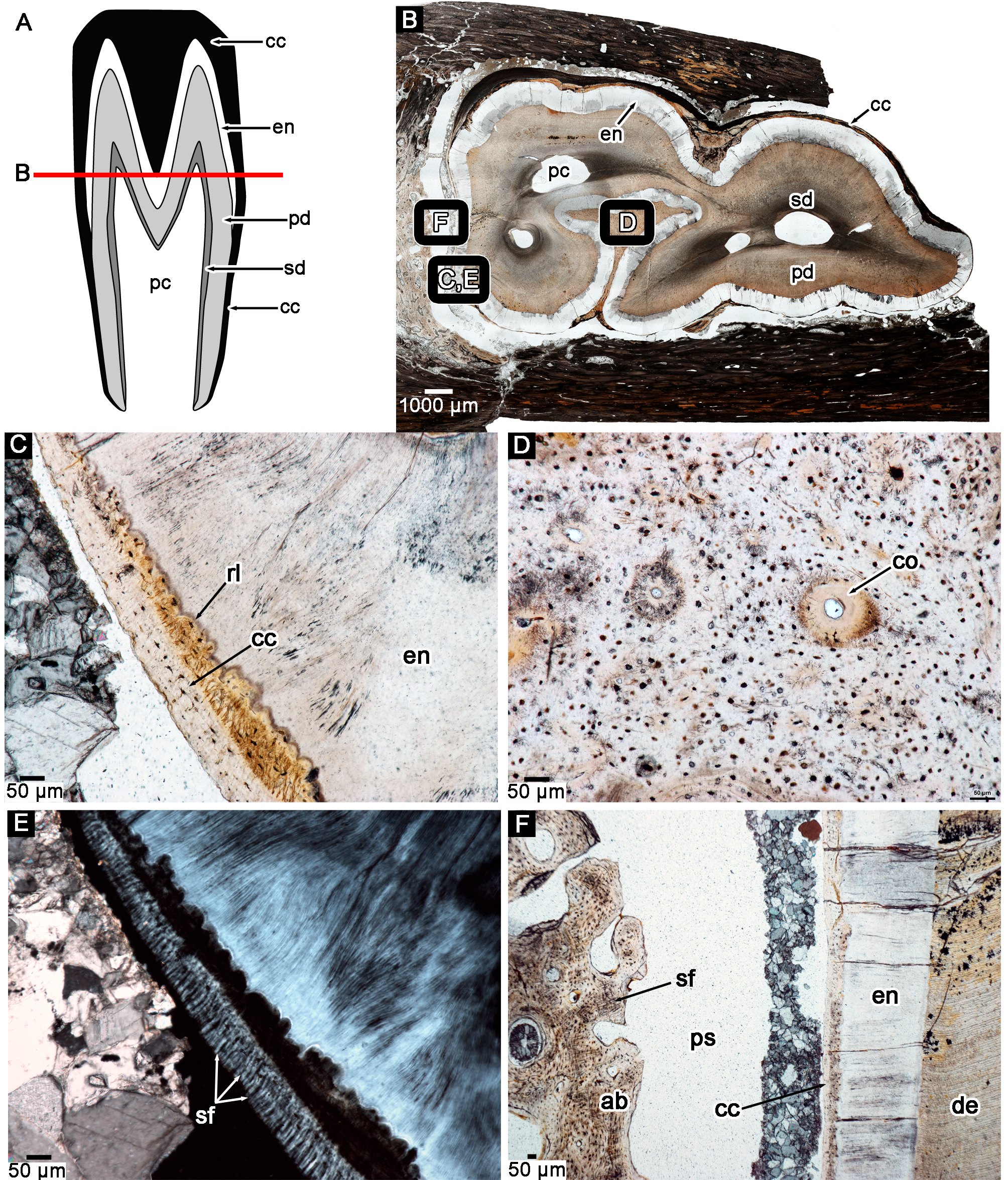

Supplement: Additional file 8: Figure S7. — Histology of the dentine and coronal cementum in a horse. (A) illustration of the major tissues in a horse tooth in longitudinal view. (B) overview image of a transverse section through a horse tooth (ROM 33036). (C) closeup image of the coronal cementum of a horse, which overlies the enamel of the tooth crown. (D) closeup image of the infundibular cementum in a horse showing abundance of cementocytes and occasional vascular spaces, which are surrounded by cementeons. (E) closeup image of coronal cementum under cross-polarized light, showing orientations of Sharpey’s fibers of the periodontal ligament. (F) closeup image of periodontal tissues of a horse. Abbrevations: ab, alveolar bone; cc, coronal cellular cementum; de, dentine; en, enamel; pc, pulp cavity; pd, primary dentine; rl, reversal line; sd, secondary dentine; sf, Sharpey’s fibers. (TIF 8893 kb) [file 12862_2016_721_MOESM8_ESM.tif]
